# Investigating spousal concordance of diabetes through statistical analysis and data mining

**DOI:** 10.1371/journal.pone.0183413

**Published:** 2017-08-17

**Authors:** Jong-Yi Wang, Chiu-Shong Liu, Chi-Hsuan Lung, Ya-Tun Yang, Ming-Hung Lin

**Affiliations:** 1 Department of Health Services Administration, China Medical University, Taichung, Taiwan; 2 Department of Family Medicine, China Medical University Hospital, Taichung, Taiwan; 3 Department of Medicine, China Medical University, Taichung, Taiwan; 4 Department of Social Work, National Quemoy University, Kinmen, Taiwan; 5 Management Center, Kuang Tien General Hospital, Taichung, Taiwan; 6 Department of Public Health, China Medical University, Taichung, Taiwan; Inselspital Universitatsspital Bern, SWITZERLAND

## Abstract

**Objective:**

Spousal clustering of diabetes merits attention. Whether old-age vulnerability or a shared family environment determines the concordance of diabetes is also uncertain. This study investigated the spousal concordance of diabetes and compared the risk of diabetes concordance between couples and noncouples by using nationally representative data.

**Methods:**

A total of 22,572 individuals identified from the 2002–2013 National Health Insurance Research Database of Taiwan constituted 5,643 couples and 5,643 noncouples through 1:1 dual propensity score matching (PSM). Factors associated with concordance in both spouses with diabetes were analyzed at the individual level. The risk of diabetes concordance between couples and noncouples was compared at the couple level. Logistic regression was the main statistical method. Statistical data were analyzed using SAS 9.4. C&RT and Apriori of data mining conducted in IBM SPSS Modeler 13 served as a supplement to statistics.

**Results:**

High odds of the spousal concordance of diabetes were associated with old age, middle levels of urbanization, and high comorbidities (all *P <* 0.05). The dual PSM analysis revealed that the risk of diabetes concordance was significantly higher in couples (5.19%) than in noncouples (0.09%; OR = 61.743, *P* < 0.0001).

**Conclusions:**

A high concordance rate of diabetes in couples may indicate the influences of assortative mating and shared environment. Diabetes in a spouse implicates its risk in the partner. Family-based diabetes care that emphasizes the screening of couples at risk of diabetes by using the identified risk factors is suggested in prospective clinical practice interventions.

## Introduction

Various studies have reported genetic factors for diabetes mellitus [1–4], warranting its familial aggregation [5–8]. Nevertheless, few studies have investigated the clustering of diabetes [9, 10], particularly in married couples who were not genetically related. A cross-sectional study on concordant diseases in couples revealed that the odds of diabetes concordance was significantly high after adjustment for age alone (odds ratio [OR] = 1.70, 95% confidence interval [CI] = 1.06–2.74) but not after adjustment for age, smoking, and body mass index (OR = 1.41, 95% CI = 0.87–2.26) [11]. The findings regarding the spousal concordance of diabetes are substantially inconclusive. Moreover, age is considered a crucial determinant of diabetes. Studies have reported that old age is strongly associated with a high risk of diabetes [4, 8, 9, 12]; the risk increases with age. Thus, middle-aged and elderly couples are susceptible to diabetes because of slowing metabolism and obesity. A common phenomenon across all the studies on the family clustering of metabolic disorders is the lack of nonfamily counterparts who did not share the same environments. Hence, it is imperative to conduct a concordance study that compares the disparity in the risk of diabetes between couples and noncouples to ascertain the effects of a common environment while examining the age vulnerability.

Most studies on family clustering have reported merely univariate statistics or investigated a very limited number of associated factors. However, familial clustering or concordance pertains to the common experiences of certain morbidities within a family and is conceivably involved with the risk factors in individual family members. Therefore, examining the factors associated with diabetes in each spouse is crucial for obtaining a more comprehensive understanding of diabetes concordance in couples. Prior research has reported sex differences in the occurrence of diabetes. Men were more likely to be diagnosed as having hyperglycemia than are women, particularly men with an older age and habits of smoking and drinking [9, 12, 13]. A study indicated no significant association between income level and diabetes prevalence [14]; however, most studies have reported an association between income and diabetes, with low household income identified as the risk factor [15, 16]. Moreover, the risk of diabetes and other metabolic syndromes varied with occupations because of varying work-related physical activities [13, 16]. Although higher levels of urbanization were associated with higher risk of diabetes [15], the association remains inconsistent. In addition, studies have indicated that diabetes could be associated with certain chronic diseases such as HIV and psychiatric morbidities [17–19]. The effects of the potential associated factors on the spousal concordance of diabetes require investigation.

Scarce studies have examined a control group and associated factors for diabetes clustering in couples. Therefore, the present study sought to determine the spousal concordance of diabetes by adopting a mathematically matched group of noncouples to compare the risk of diabetes concordance between couples and noncouples by using nationally representative data.

## Methods

### Hypothesis and research design

This study hypothesized that the risk of spousal concordance of diabetes is associated with the individual and shared characteristics of spouses. The individual characteristics of spouses may exert different effects on spousal concordance of diabetes. Moreover, the study hypothesized the existence of a disparity in the risk of concordance between couples and noncouples. The two hypotheses were tested in a longitudinal population-based cohort by using a case–control design. The study was approved by the Research Ethics Committee of China Medical University Hospital, Taiwan.

### Data source and study sample

The National Health Insurance (NHI) program, established in 1995, provides comprehensive health care benefits to more than 99.7% of the residents of Taiwan (N = 23.50 million). All the medical claims from this universal program are managed by the National Health Research Institutes (NHRI), which releases the population-based National Health Insurance Research Database (NHIRD). This retrospective study retrieved longitudinal data from the 2002–2013 registry of the NHIRD, which contains the reimbursement claims of 1 million randomly sampled beneficiaries. The NHRI has indicated that this NHIRD subset can completely represent all the enrollees. The claim diagnoses in the NHIRD were coded using the International Classification of Diseases, Ninth Revision, Clinical Modification (ICD-9-CM).

This study used the data fields “relation” and encrypted individual identifiers to match married spouses from the NHIRD registry. Only two individuals having a relationship status of being insured and dependent spouses were identified as a couple by using “spouse” in the data field “relation” and the prerequisite of the encrypted identifiers mutually matched between the two spouses. Furthermore, to obtain an initial diagnosis of diabetes throughout the observation period, individuals diagnosed as having diabetes mellitus (ICD-9-CM 250.x) in 2002 were excluded from the study. Patients younger than 16 years were also excluded. Initially, data of 5,680 couples were obtained. However, 43 patients were excluded because of inadequate or missing data (0.76%). Consequently, the current study identified a cohort of 5,643 married couples, comprising 11,286 individuals (5,643 insured and 5,643 dependent spouses).

To ascertain the similarity between the case (couples) and control (noncouples) groups, except for the couple status, the case group was matched with the control group in terms of the same single value of sex, age, and comorbidities through 1:1 propensity score matching (PSM) to reduce selection bias [20]. This procedure was repeated twice for each member of a couple to obtain two randomly selected noncouple counterparts (total four individuals in the matching: dual PSM). Thus, the three matched variables were tested twice for any significant differences between the two groups. The results indicated high similarity with no differences in sex, age, or comorbidities (all *P* = 1, Table 1), thus confirming that the couples and noncouples qualified for the comparison. PSM provides an alternative to adjust for covariates at the level of multivariate analysis [21]. Consequently, 5,643 couples and 5,643 noncouples (N = 22,572 individuals) were included in the subsequent analysis.

**Table 1:** Comparisons of characteristics after 1:1 dual propensity score matching for couples and noncouples (N = 11,286 pairs).

Variables ^1^	Insured spouse		Counterpart of insured spouse		χ^2^ *P*-value	Dependent spouse		Counterpart of dependent spouse		χ^2^ *P*-value
	n_1_	%	n_2_	%		n_3_	%	n_4_	%	
*Sex*					1.0000					1.0000
Female	1,460	25.87	1,460	25.87		4,183	74.13	4,183	74.13	
Male	4,183	74.13	4,183	74.13		1,460	25.87	1,460	25.87	
*Age*					1.0000					1.0000
16–44 years	1,674	29.67	1,674	29.67		2,089	37.02	2,089	37.02	
45–54 years	1,607	28.48	1,607	28.48		1,391	24.65	1,391	24.65	
55–64 years	1,185	21.00	1,185	21.00		1,152	20.41	1,152	20.41	
≥ 65 years	1,117	20.86	1,117	20.86		1,011	17.92	1,011	17.92	
*Comorbidity (CCI)*					1.0000					1.0000
0	3,853	68.47	3,853	68.47		4,102	72.91	4,102	72.91	
1	1,575	27.99	1,575	27.99		1,329	23.62	1,329	23.62	
2	111	1.97	111	1.97		114	2.03	114	2.03	
≥ 3	88	1.56	88	1.56		81	1.44	81	1.44	

^1^Sex, age, and comorbidities were matched twice for a total of four individuals of couples and noncouples.

### Variables

The concordance of diabetes was determined using a dichotomous outcome variable. Concordance was reported if both spouses or counterparts were diagnosed using ICD-9-CM codes (250.x) for diabetes mellitus; otherwise, discordance was reported.

The present study included two categories of independent variables that are possibly associated with diabetes: 1. characteristics of the insured spouse, comprising sex, age, premium-based monthly salary, occupation, urbanization level, region, catastrophic illness or injury, and comorbidities; and 2. characteristics of the dependent spouse, comprising age, catastrophic illness or injury, and comorbidities. The urbanization level and region were considered common environmental characteristics of the couples. The remaining variable was the characteristics of the individual spouses. Legally, the Taiwan government allows only heterosexual marriage; thus, one sex, that of the insured spouse, was used to eliminate collinearity. Age did not pass the normality test, including skewness and kurtosis, and was therefore classified into five ordinal levels, according to the frequency distribution. Furthermore, premium-based monthly salary, occupation, region, and catastrophic illness or injury were defined on the basis of the official NHI classifications. The National Health Insurance Administration issues the Catastrophic Illness and Injury card to patients with severe illness or injury. Patients with numerous catastrophic illness and injury conditions, such as regular dialysis or permanent disability, can apply for the card after the severity reaches the official criteria of the NHI program and is verified by a board-certified physician. Comorbidities were assessed using the Charlson comorbidity index (CCI) [22], a frequently used measure in clinical research. After original scoring from 0 to 6 conducted by weighting ICD-9-CM codes for each spouse, this study classified comorbidities into 0 (no comorbidities) and 1–3 (high comorbidities) because of the low-frequency distribution of CCI scores exceeding 3. The urbanization level was graded using a 5-point scale, with 1 and 5 indicating the highest and lowest urbanization levels, respectively. All the 11 independent variables were measured on a categorical or an ordinal level. All the variables in the case–control design were defined at the pair level (couples versus noncouples).

### Data analysis

In this study, data were analyzed through statistical analysis and data mining. Statistical methods included the chi-squared test and logistic regression. The chi-squared test determined the prevalence rates of diabetes concordance at the bivariate level. Logistic regression was used mainly for predicting diabetes concordance at the multivariate level, with the adjusted odds ratio (OR) and corresponding 95% confidence interval (CI). Because the members of the couples and noncouples were matched for the three variables, conditional logistic regression was used to analyze the matched pair data without the matching factors in the regression model [23, 24]. The conditional likelihood was estimated within the same matched set for binary diabetes concordance [25]. Moreover, collinearity diagnostics were computed using indices including variance inflation and tolerance. For data mining, C&RT and Apriori, two methods under the no hypothesis paradigm, were used to explore hidden patterns that statistics might fail to detect [26, 27]. The application of data mining techniques in longitudinal study analysis of a large clinical data source may discover useful information on disease prediction and health care delivery [28–30]. C&RT, a decision tree, was used for classification [31]. The Apriori algorithm of association rules was used to mine for potential associations in the extracted research data [32]. Data mining largely served as a supplement to statistics. In contrast to theory-based statistical analysis, data mining is substantially more data-driven. Research that analyzes the individual level factors associated with the couple concordance of diabetes is still lacking. Therefore, this study used statistics and data mining for the optimization of pioneering modeling for the concordance factors. The joint findings engendered by the two approaches should increase the strength of evidence on diabetes concordance. Data were analyzed using SAS 9.4 and IBM SPSS Modeler 13.

## Results

The common characteristics of 11,286 individual spouses were analyzed and merged in the unit of a couple. Table 2 lists the descriptive statistics of the 5,643 couples, including age and health characteristics. Most couples were aged 16–44 years (33.34%), without catastrophic illness or injury (91.29%) or any comorbidities (70.69%). A summary of cross‐tabulations of the three characteristics and sex is listed in Table 2. Table 3 presents the characteristics of spouses and their associations with spousal concordance of diabetes. The prevalence rates of diabetes in the insured and dependent spouses were 18.41% (1,039/5,643) and 16.64% (939/5,643), respectively. When calculated in the unit of one couple, the prevalence rate of diabetes in either the insured or dependent spouse of the 5,643 couples was 24.67% (1,392/5,643); however, only 16.92% of the noncouples included one individual diagnosed as having diabetes (n = 955). The cross-tabulations of individual spouse characteristics and diabetes in only one spouse of a couple are presented as the intermediate results of concordance. Furthermore, the chi-squared test revealed that nine independent variables were significantly associated with spousal concordance: age, monthly income, occupation, region, catastrophic illness or injury, and comorbidities of the insured spouse, as well as age, catastrophic illness or injury, and comorbidities of the dependent spouse (all *P <* 0.0001). Overall, old age (≥65 years), low monthly income (≤US$760), catastrophic illness or injury, and CCI = 2 were significantly associated with a higher prevalence of spousal concordance. Insured spouses who were soldiers, social security insured, veterans, and associated with religious groups were more likely to develop spousal concordance of diabetes, compared with those involved in other occupations. This study did not detect any signs of collinearity.

**Table 2:** Characteristics of the study couples during 2002–2013 (N = 5,643 couples ^1^).

Variables	Frequency	%	Male		Female	
			n_1_	%_1_	n_2_	%_2_
*Age*						
16–44 years	3,763	33.34	1579	41.96	2184	58.04
45–54 years	2,998	26.56	1566	52.23	1432	47.77
55–64 years	2,337	20.71	1201	51.39	1136	48.61
≥ 65 years	2,188	19.39	1297	59.28	891	40.72
*Catastrophic illness or injury*						
Absent	10,303	91.29	5081	49.32	5222	50.68
Present	983	8.71	562	57.17	421	42.83
*Comorbidity (CCI)*						
0	7,955	70.69	3828	48.12	4127	51.88
1	2,904	25.81	1587	54.65	1317	45.35
2	225	2.00	121	53.78	104	46.22
≥ 3	169	1.50	87	51.48	82	48.52

^1^11,286 members of the couples were analyzed.

**Table 3:** Chi-squared test for spousal concordance of diabetes during 2002–2013 (N = 5,643 couples).

Variables	Frequency	%	No diabetes		One spouse with diabetes ^1^		Spousal concordance		χ^2^ *P*-value
			n_1_	%_1_	n_2_	%_2_	n_3_	%_3_	
Total			3,958	70.14	1,392	24.67	293	5.19	
**Insured Spouse Characteristics**									
*Sex*									0.4687
Female	1,460	25.87	1,032	70.68	346	23.70	82	5.62	
Male	4,183	74.13	2,926	69.95	1,046	25.01	211	5.04	
*Age*									< .0001*
16–44 years	1,674	29.67	1,449	86.56	214	12.78	11	0.66	
45–54 years	1,607	28.48	1,253	77.97	313	19.48	41	2.55	
55–64 years	1,185	21.00	742	62.62	371	31.31	72	6.08	
≥ 65 years	1,177	20.86	514	43.67	494	41.97	169	14.36	
*Premium-based monthly salary (USD$)*									< .0001*
≤ 760	3,629	64.31	2,445	67.37	963	26.54	221	6.09	
760–960	413	7.32	294	71.19	96	23.24	23	5.57	
960–1210	481	8.52	357	74.22	106	22.04	18	3.74	
1210–1526	473	8.38	367	77.59	95	20.08	11	2.33	
> 1526	647	11.47	495	76.51	132	20.40	20	3.09	
*Occupation*									< .0001*
First category (Private employee and government employee)	2,884	51.11	2,162	74.97	622	21.57	100	3.47	
Second category (Labor union member)	785	13.91	556	70.83	196	24.97	33	4.20	
Third category (Farmer and Fisherman)	695	12.32	454	65.32	189	27.19	52	7.48	
Fourth, fifth, and sixth categories (Soldier, social security insured, and veteran and religious group member)	1,279	22.67	786	61.45	385	30.10	108	8.44	
*Urbanization level*									0.6519
Level 1 (Highest)	1,745	31.04	1,226	70.26	415	23.78	104	5.96	
Level 2	1,666	29.63	1,161	69.69	423	25.39	82	4.92	
Level 3	953	16.95	682	71.56	232	24.34	39	4.09	
Level 4	739	13.14	513	69.42	186	25.17	40	5.41	
Level 5 (Lowest)	519	9.23	364	70.13	129	24.86	26	5.01	
*Region*									< .0001*
Taipei	1,980	35.17	1,397	70.56	477	24.09	106	5.35	
Northern	869	15.44	615	70.77	200	23.01	54	6.21	
Central	1,076	19.12	791	73.51	245	22.77	40	3.72	
Southern	752	13.36	487	64.76	215	28.59	50	6.65	
Southeast	847	15.05	598	70.60	209	24.68	40	4.72	
Eastern	105	1.87	63	60.00	41	39.05	1	0.95	
*Catastrophic illness or injury*									< .0001*
Absent	5,106	90.48	3,676	71.99	1,191	23.33	239	4.68	
Present	537	9.52	282	52.51	201	37.43	54	10.06	
*Comorbidity (CCI)*									< .0001*
0	3,853	68.47	2,973	77.16	772	20.04	108	2.80	
1	1,575	27.99	875	55.56	544	34.54	156	9.90	
2	111	1.97	51	45.95	42	37.84	18	16.22	
≥ 3	88	1.56	45	51.14	32	36.36	11	12.50	
**Dependent Spouse Characteristics**									
*Age*									< .0001*
16–44 years	2,089	37.02	1,796	85.97	276	13.21	17	0.81	
45–54 years	1,391	24.65	1,046	75.20	304	21.85	41	2.95	
55–64 years	1,152	20.41	666	57.81	398	34.55	88	7.64	
≥ 65 years	1,011	17.92	450	44.51	414	40.95	147	14.54	
*Catastrophic illness or injury*									< .0001*
Absent	5,197	92.10	3,706	71.31	1,254	24.13	237	4.56	
Present	446	7.90	252	56.50	138	30.94	56	12.56	
*Comorbidity (CCI)*									< .0001*
0	4,102	72.91	3,135	76.43	843	20.55	124	3.02	
1	1,329	23.62	709	53.35	479	36.04	141	10.61	
2	114	2.03	55	48.25	40	35.09	19	16.67	
≥ 3	81	1.44	45	55.56	27	33.33	9	11.11	

^1^Either insured spouse or dependent spouse per one couple; averaged 24.67% > 16.92% in one individual with diabetes of noncouples. Row percentages are presented.

* *P* < 0.05.

Table 4 presents the logistic regression results. The results of the unadjusted model indicated that 10 independent variables were significantly associated with spousal concordance (all *P* < 0.05). After all other covariates were held constant, nine variables remained significantly associated with spousal concordance of diabetes (all *P* < 0.05). Male insured spouses were more likely to experience spousal concordance than their female counterparts were (OR = 1.587; 95% confidence interval [CI] = 1.181–2.133). Insured spouses aged 45–54, 55–64, and ≥65 years were more likely to experience spousal concordance (OR = 3.817, 8.084, and 17.127; 95% CI = 1.950–7.472, 4.224–15.473, and 8.962–32.732; respectively), compared with those aged 16–44 years. Moreover, insured spouses residing in areas with urbanization levels of 2 and 3 were more likely to experience spousal concordance (OR = 1.425 and 1.817; 95% CI = 1.004–2.021 and 1.167–2.828; respectively), compared with those in level 1 urbanization areas. The odds of spousal concordance were significantly lower in insured spouses residing in the northern region than those residing in Taipei (OR = 0.632; 95% CI = 0.420–0.951). Regarding health characteristics, the odds of spousal concordance were significantly higher in insured spouses with catastrophic illness or injury than in those without these factors (OR = 1.527; 95% CI = 1.004–2.001). The odds of spousal concordance were significantly higher in insured spouses with medium–high comorbidity (CCI = 2) than in those without comorbidities (OR = 1.556; 95% CI = 1.009–3.618). Dependent spouses aged 45–54, 55–64, and ≥65 years were more likely to experience spousal concordance (OR = 3.405, 8.338, and 13.882; 95% CI = 1.921–6.035, 4.895–14.201, and 8.162–23.609; respectively), compared with those aged 16–44 years. Moreover, dependent spouses with catastrophic illness or injury were more likely to experience spousal concordance (OR = 1.478; 95% CI = 1.005–2.071), compared with those without these factors. In addition, dependent spouses with medium–high comorbidity (CCI = 2) were more likely to experience spousal concordance (OR = 1.904; 95% CI = 1.453–2.496), compared with those without comorbidities.

**Table 4:** Logistic regression models for spousal concordance during 2002–2013 (N = 5,643 couples).

Variables	Bivariate model			Multivariate model		
	Crude OR	95% CI	*P*-value	Adjusted OR	95% CI	*P*-value
**Insured Spouse Characteristics**						
*Sex*						
Female	—	—	—	—	—	—
Male	1.120	0.862–1.456	0.3964	1.587	1.181–2.133	0.0022*
*Age*						
16–44 years	—	—	—	—	—	—
45–54 years	3.958	2.027–7.728	< .0001*	3.817	1.950–7.472	< .0001*
55–64 years	9.780	5.162–18.529	< .0001*	8.084	4.224–15.473	< .0001*
≥ 65 years	25.347	13.705–46.877	< .0001*	17.127	8.962–32.732	< .0001*
*Premium-based monthly salary (USD$)*						
≤ 760	—	—	—	—	—	—
760–960	1.100	0.707–1.711	0.6738	0.814	0.481–1.377	0.4427
960–1210	1.668	1.022–2.723	0.0408*	1.117	0.626–1.994	0.7078
1210–1526	2.723	1.475–5.026	0.0014*	1.584	0.793–3.161	0.1924
> 1526	2.033	1.276–3.238	0.0028*	1.271	0.712–2.269	0.4174
*Occupation*						
First category (Private employee and government employee)	—	—	—	—	—	—
Second category (Labor union member)	0.819	0.548–1.223	0.3285	1.106	0.693–1.766	0.6714
Third category (Farmer and Fisherman)	0.444	0.314–0.628	< .0001*	0.874	0.532–1.437	0.5965
Fourth, fifth, and sixth categories (Soldier, social security insured, and veteran and religious group member)	0.389	0.294–0.516	< .0001*	0.737	0.487–1.117	0.1500
*Urbanization level*						
Level 1 (Highest)	—	—	—	—	—	—
Level 2	1.224	0.909–1.649	0.1826	1.425	1.004–2.021	0.0472*
Level 3	1.485	1.019–2.164	0.0397*	1.817	1.167–2.828	0.0082*
Level 4	1.108	0.761–1.612	0.5937	1.365	0.857–2.174	0.1908
Level 5 (Lowest)	1.202	0.773–1.869	0.4144	1.618	0.922–2.841	0.0935
*Region*						
Taipei	—	—	—	—	—	—
Northern	0.854	0.609–1.197	0.3588	0.632	0.420–0.951	0.0278*
Central	1.465	1.010–2.124	0.0440*	1.188	0.783–1.802	0.4188
Southern	0.794	0.561–1.124	0.1933	0.681	0.445–1.041	0.0758
Southeast	1.141	0.786–1.657	0.4877	1.077	0.711–1.629	0.7271
Eastern	5.879	0.813–42.514	0.0793	6.420	0.863–47.770	0.0694
*Catastrophic illness or injury*						
Absent	—	—	—	—	—	—
Present	2.277	1.671–3.104	< .0001*	1.527	1.004–2.001	< .0001*
*Comorbidity (CCI)*						
0	—	—	—	—	—	—
1	1.129	0.514–2.644	0.2785	1.094	0.547–2.188	0.7985
2	1.685	1.342–2.842	< .0001*	1.556	1.009–3.618	0.0009*
≥ 3	1.603	1.174–3.744	0.0009*	1.422	0.817–2.475	0.3046
**Dependent Spouse Characteristics**						
*Age*						
16–44 years	—	—	—	—	—	—
45–54 years	3.702	2.094–6.542	< .0001*	3.405	1.921–6.035	< .0001*
55–64 years	10.080	5.966–17.032	< .0001*	8.338	4.895–14.201	< .0001*
≥ 65 years	20.737	12.473–34.475	< .0001*	13.882	8.162–23.609	< .0001*
*Catastrophic illness or injury*						
Absent	—	—	—	—	—	—
Present	3.005	2.206–4.093	< .0001*	1.478	1.005–2.071	0.0232*
*Comorbidity (CCI)*						
0	—	—	—	—	—	—
1	1.289	0.694–2.394	0.4217	1.081	0.824–1.417	0.5737
2	2.367	1.929–2.904	< .0001*	1.904	1.453–2.496	< .0001*
≥ 3	1.319	1.142–2.711	0.0013*	1.188	0.783–1.802	0.4188

* *P* < 0.05. OR: odds ratio

Table 5 presents the results of couple-level analysis following 1:1 dual PSM. The chi-squared test revealed a significant association of marital status with diabetes concordance (*P* < 0.0001). Couples were significantly associated with a higher prevalence of concordance (5.19% versus 0.09%) than were noncouples. The percentage of one spouse diagnosed with diabetes in couples was higher than that of one individual with diabetes in noncouples. This phenomenon is consistent among both male and female (18.54% > 13.38%, 6.13% > 3.54%, respectively). Moreover, conditional logistic regression indicated that marital status was significantly associated with diabetes concordance (*P* < 0.0001). The odds of diabetes concordance were significantly higher in couples than in noncouples (OR = 61.743; 95% CI = 26.128–191.726).

**Table 5:** Concordance of diabetes in couples and noncouples (dual propensity score matched for sex, age, and comorbidities; chi-squared test and conditional logistic regression; N = 11,286 pairs).

Variables	No diabetes		Only male		Only female		Concordance of diabetes		χ^2^ *P*-value	Concordance of diabetes		
	n_1_	%_1_	n_2_	%_2_	n_3_	%_3_	n_4_	%_4_		OR	95% CI	*P*-value
***Relation***									< .0001*			
Non-couple	4,683	82.99	755	13.38	200	3.54	5	0.09		—	—	—
Couple	3,958	70.14	1,046	18.54	346	6.13	293	5.19		61.743	26.128–191.726	< .0001*

* *P* < 0.05. OR: odds ratio

After feature selection, data mining was performed with a reduced set of relevant data. The following classification rules were identified for predicting spousal concordance: 1. CCI ≥ 1; fourth, fifth, and sixth categories of occupation; and residence in northern and southern regions for insured spouses; and 2. age ≥ 55 years and CCI ≥ 1 for dependent spouses. For predicting no spousal concordance, the classification rules were a monthly income of ≥US$960 and no comorbidities for insured spouses and age = 16–54 years and no comorbidities for dependent spouses. The prediction accuracy of C&RT was 85.7%–90.9%. The Apriori algorithm was not sensitive in detecting the association rules for the presence of spousal concordance. However, the acquired rules for predicting no spousal concordance included the male sex, age = 16–44 years, no catastrophic illness or injury, and no comorbidities for insured spouses, as well as age = 16–44 years and no catastrophic illness or injury for dependent spouses. Confidence in Apriori is an indication of the probability that the rule is correct. In this study, the confidence of the Apriori algorithm was 95.3%–98.2%, indicating a strong association between the extracted patterns and spousal concordance of diabetes. Overall, the indices of accuracy and confidence demonstrate effective data mining [33, 34].

## Discussion

### High concordance in couples versus low concordance in noncouples

To our knowledge, this study is the first that investigated spousal concordance of diabetes in a matched case-control design. A contrast of high and low concordance rates of diabetes in couples and noncouples, respectively, was identified. The dual PSM analysis revealed this phenomenon in both prevalence rates and ORs. The determined prevalence rate of spousal concordance was 5.19% (293/5,643) in couples, strongly higher than in noncouples (0.09%). The OR of 61.743 represents the marked effect of a common family environment on the development of diabetes in couples and deserves emphasis.

Both couples and noncouples were matched by sex, age, and comorbidities; therefore, the high contrast in the concordance is not attributable to old-age vulnerability and is closely related only to the coupled status. Assortative mating and similarities between both members of a married couple in a common environment may explain the high concordance of diabetes in couples [35]. Studies have indicated resemblances between spouses [36, 37], particularly in long-standing couples. Notably, collectivism in Taiwanese culture [38] may reinforce behavioral resemblances in couples. Furthermore, through cohabitation in the same family environment, concordant health behaviors, including exercise and dietary habits, and shared lifestyles in couples can be shaped [39–42] and might thus lead to a shared exposure, such as concordant obesity [43], to diabetes [44]. Hence, family-based intervention for modifiable health behaviors is a priority in clinical practice.

### Individual-level characteristics predicting couple-level concordances

Statistical analysis and data mining yielded the combined results regarding factors associated with spousal concordance of diabetes. In addition to the couple status, nine factors, including personal and shared characteristics, of spousal concordance warrant attention. Most insured spouses were men who could have a higher risk of diabetes than their female counterparts [9, 23]. The prevalence rate of diabetes was higher in insured spouses (18.41% in insured spouses versus 16.64% in dependent spouses), thus explicating the finding that insured men were more likely to experience spousal concordance of diabetes than were insured women (Table 4). Old age was markedly associated with high risks of concordant diabetes, particularly in spouses aged ≥65 years (both ORs > 13, accuracy = 85.7%–90.9%); this observation is in accordance with the findings of previous studies [45, 46]. The urbanization level and region, which are the shared geographical characteristics of couples, were identified as the determinants of spousal concordance. Levels 2 and 3 of urbanization were associated with higher odds of spousal concordance, whereas residence in the northern region was associated with a lower risk. The geographical disparities in concordant diabetes warrant further research and require the attention of health policy-makers. The findings regarding comorbidities are similar to those previously reported [47, 48] and indicate that medical conditions of individual spouses contribute to concordant diabetes in couples.

Overall, diabetes in a spouse may indicate the risk of diabetes in the partner. A previous study indicated that spousal diabetes is associated with a 26% increase in the risk of diabetes in the partner [49], echoing the present findings. The phenomenon of spousal concordance of diabetes is evident. Therefore, the clinical prevention of diabetes should target spouses whose married partners were diagnosed as having diabetes by applying the individual-level and shared geographical risk factors identified in this study, including old age, mid-range urbanization, and chronic morbidities.

### Couple-oriented health insurance: couplitation

Health insurance schemes might adjust medical payments by sex, age, and morbidities, such as capitation reimbursement [50]. A family history of certain chronic and catastrophic illnesses among genetically related family members is considered for determining premiums. Nevertheless, the spouse history of diabetes is typically not involved in the risk rating of individual-level health insurance plans. Therefore, the present study proposes a novel yet reasonable direction of a couple-oriented insurance scheme, couplitation, that is aimed at developing comprehensive coverage and reimbursements for spouse-vulnerable chronic diseases [51–53], particularly diabetes. Couplitation may improve early detection through examination in a manner paralleling capitation. This spouse-related risk rating of an insurance scheme requires feasibility analysis in future studies.

The limitations of the present study are mainly related to the database used. First, the NHIRD does not include information on the educational level, health behaviors, laboratory test results, cohabitation duration, and other joint characteristics of the couples. The absence of these data weakens the statistical strength of this study. Second, the body mass index is a major risk factor for diabetes; the absence of this factor may result in residual confounding and thus bias the findings in an unknown direction. Third, high level of awareness or knowledge of symptoms of diabetes may lead to early diagnosis. Due to the lack of awareness-related data in the NHIRD, the current study failed to take this factor into consideration. Finally, all spouses retrieved from the database were limited to the insured–dependent relationship. The generalization of the study findings to all other relationships requires deliberation.

## Conclusions

This study involved cohort and case–control designs, individual- and couple-level analyses, and statistical analysis and data mining, all of which were aimed at providing strong evidence. This study adds to the existing knowledge base by determining the evident effects of a common family environment and individual characteristics on diabetes concordance in couples. Old-age vulnerability in diabetes cannot explain this high concordance phenomenon in couples. Diabetes in one spouse indicates the risk of diabetes in the partner. Therefore, this study suggests that family-based diabetes health care and clinical intervention be conducted using the individual risk factors identified in this study. Future studies may focus on investigating the spousal concordance of a specific type of diabetes.
